# A new unique form of microRNA from human heart, microRNA-499c, promotes myofibril formation and rescues cardiac development in mutant axolotl embryos

**DOI:** 10.1186/1423-0127-20-20

**Published:** 2013-03-23

**Authors:** Andrei Kochegarov, Ashley Moses, William Lian, Jessica Meyer, Michael C Hanna, Larry F Lemanski

**Affiliations:** 1 Department of Biological and Environmental Sciences, Texas A&M University-Commerce, P.O. Box 3011, Commerce, TX 75429-3011, USA

**Keywords:** Myofibrillogenesis, Tropomyosin, Cardiac mutant axolotl, Human embryonic heart RNA, microRNA

## Abstract

**Background:**

A recessive mutation “c” in the Mexican axolotl, *Ambystoma mexicanum*, results in the failure of normal heart development. In homozygous recessive embryos, the hearts do not have organized myofibrils and fail to beat. In our previous studies, we identified a noncoding Myofibril-Inducing RNA (MIR) from axolotls which promotes myofibril formation and rescues heart development.

**Results:**

We randomly cloned RNAs from fetal human heart. RNA from clone #291 promoted myofibril formation and induced heart development of mutant axolotls in organ culture. This RNA induced expression of cardiac markers in mutant hearts: tropomyosin, troponin and α-syntrophin. This cloned RNA matches in partial sequence alignment to human microRNA-499a and b, although it differs in length. We have concluded that this cloned RNA is unique in its length, but is still related to the microRNA-499 family. We have named this unique RNA, microRNA-499c. Thus, we will refer to this RNA derived from clone #291 as microRNA-499c throughout the rest of the paper.

**Conclusions:**

This new form, microRNA-499c, plays an important role in cardiac development.

## Background

The Mexican axolotl, *Ambystoma mexicanum*, is an exciting and useful animal model to study vertebrate heart development and cardiac myofibrillogenesis. It carries a lethal cardiac recessive mutation, designated by gene “c”, which, when homozygous (c/c), prevents normal heart development in axolotl embryos. Our previous studies [1] have shown that a non-coding RNA, Myofibril-Inducing RNA (MIR) from normal axolotl, is capable of promoting myofibrillogenesis and beating hearts in the mutant (c/c) axolotl embryos. This study demonstrated that the MIR gene is essential for tropomyosin expression in axolotl hearts during development. Real-Time PCR studies showed that mRNA expression of various tropomyosin isoforms in untreated mutant hearts is similar to normal hearts knocked down with double-stranded MIR (dsMIR). These results suggest that MIR is involved in controlling expression of various tropomyosin isoforms and subsequently in regulating cardiac contractility. The MIR was sequenced and found to be 166 nucleotides in length [2]. This RNA is unique in that it does not show significant homology to any known sequences in the online NCBI database. Comparison between MIR sequences obtained from normal and mutant embryos demonstrated a single point mutation at base 93 of the mutant MIR nucleotide sequence [2]. Genebee (Moscow State University), an online bioinformatics tool for the computation and modeling of secondary structures of RNAs, showed a conformational difference between the normal bioactive MIR and the mutant MIR, suggesting that the secondary structure of MIR might be important in the mutant rescue process [2]. More recently we have found that total human fetal heart RNA also has the ability to promote normal myofibril formation and restore function of the mutant axolotl hearts, suggesting that a functional homologue of the axolotl MIR could be present in human fetal heart tissue [3]. In the present study, through the random cloning of genes expressed in human heart, we have found a clone with the capacity to produce RNAs with the capability of promoting myofibrillogenesis and rescuing cardiac mutant axolotl hearts similarly to the axolotl MIR. We have designated this clone as a microRNA-499c. The role of microRNA-499 in heart development in human cardiomyocyte differentiation was previously described [4,5]. Our identification of the human microRNA which promotes myofibrillogenesis helps us to understand the molecular mechanism of heart development and may have important implications for future treatment of myocardial infarcts, cardiomyopathies and other congenital or acquired myocardial diseases in humans.

## Methods

### Cloning

For cloning, a total of 2 μg of human fetal heart RNA (Agilent Technologies, Inc #540165) was used for each reaction. The cloning kit used was the CloneMiner™ II cDNA Library Construction Kit (Invitrogen, #A11180). First and second DNA strands were synthesized from template RNAs and ligated into the pDONR222 vector. The pDONR222 vector contains the kanamycin resistance gene which allows selection of transfected bacteria and the ccdB gene which interferes with *E. coli* DNA gyrase allowing negative selection of the donor vector in *E. coli* following recombination and transformation. The ElectroMAX™ DH10B™ T1 Phage Resistant *E. coli* strain provided with the kit was transformed using the EC 1000 Electroporator (Thermo ES) at 2800 V. To each sterile cuvette, 50 μl ElectroMAX™ DH10B cells, 1.5 μl of (150 ng/μl) vector and 50 μl of dH_2_O were added. In case the sample arced at this voltage setting, 100 μl of dH_2_O, or more, was added to increase electrical resistance. After electroporation, the cells were added to 1 ml of S.O.C. medium and cultured in 15 ml snap-cap tubes for at least 1 hour at 37°C on a shaker at 225–250 rpm to allow expression of the kanamycin resistance marker. Serial dilutions of sample aliquots with S.O.C. medium at the ratios 1:10, 1:100 and 1:1000 were plated on LB agar plates containing 50ug/ml of kanamycin. The remaining cells were frozen at -80°C. Plated cells were incubated overnight at 37°C. Individual colonies were collected and transferred into snap-cap tubes with 2 ml of 2xYT medium containing 50ug/ml of kanamycin and incubated overnight. Plasmids with clones were extracted according to the standard Miniprep Plasmid DNA Isolation Protocol found in the online archive of the Institute of Bioinformatics and Applied Biotechnology.

### BsrGI digestion

Extracted plasmids (5 μl sample) were digested by 20U (1U/μl) of enzyme BsrGI in 1X NE Buffer with 0.1 mg/μl of BSA. The mixtures were incubated for 1 h at 37°C and analyzed by Gel electrophoresis on 1% agarose gels containing 0.5 μg/ml of ethidium bromide.

### PCR

T7 RNA polymerase binding site TAATACGACTCACTATAGGG was added to the 5^′^ end of forward and reverse M13 primers.

Forward primer: 5^′^-TAATACGACTCACTATAGGGGTAAAACGACGGCCAG-3^′^.

Reverse primer 5^′^-TAATACGACTCACTATAGGGCAGGAAACAGCTATGAC-3^′^.

PCR was performed using a MyTaq™ Red Mix kit (Bioline, BIO-25043) according to the instruction manual for this kit: denaturation at 95°C during 15 sec followed by annealing at 55°C for 15 sec and elongation at 72°C for 15 sec for 30 cycles. The resulting DNA was purified by 5 M sodium chloride salt and isopropanol precipitation. Pellets were washed with 70% ethanol and re-suspended in 1X Tris-EDTA buffer.

### RNA synthesis

The transcription reaction mixture was assembled from the MAXIscript® T7 Kit, Ambion # AM1314M. Then, we added 1 μg of DNA from the PCR product, 2 μL of 10X transcription buffer, 2 μL of T7 Enzyme Mix and 1 μL of each (10 mM) NTP; and adjusted the volume to 20 μL by nuclease-free water. The reaction mixture was incubated at 37°C for 2 hours. RNA was purified using ammonium acetate and ethanol precipitation and resuspended in nuclease-free water. The concentration of RNA was determined spectrophotometrycally at 260 nm.using a Synergy HT (Bio-Tek) platereader.

### Bioassay

Cardiac mutant non-function carrier (+/c) adult axolotls were obtained from the Ambystoma Genetic Stock Center, University of Kentucky, Lexington. These heterozygous adult animals were mated (+/c x +/c) to produce mutant (c/c) and wildtype (+/+) embryos for our studies. Embryos were collected and allowed to develop to heart-beat stages 35-36, according to the Bordzilovskaya et al. staging system [6]. For bioassays, only double recessive mutant c/c embryos were selected which do not have beating hearts. The embryos were anaesthetized by 0.7 mg/ml tricaine methanesulfonate or Ms-222 (Argeitt Chemicals Labs) in Holtfreter’s solution [7]. Embryos were dissected under a binocular microscope in clay-lined Petri dishes in Holtfreter’s medium containing 1% antibiotic/antimycotic (Gibco #15240). Hearts were transferred into the Petri dishes on Parafilm substrate into 50 μl of Holtfreter’s solution (without antibiotic) containing 7 ng/μl of human fetal heart RNA from individual clones along with 0.1 mg/ml of lipofectamine^.^ reagent (Invitrogen, Carlsbad CA). The Petri dishes with hearts were enclosed in a plastic container containing wet paper towels to maintain a saturated humidity environment at 17°C.

### Fixation and staining procedure

All steps were performed at room temperature as previously described [2]. Hearts were fixed in 4% paraformaldehyde for 30 min and rinsed twice in PBS for 3 min. Hearts were permeabilized in 0.05% Tween-20 and 3% BSA in PBS for 1 h. Hearts were incubated overnight with monoclonal anti-tropomyosin CG3 antibody (Developmental Studies Hybridoma Bank, University of Iowa) diluted to 1:75 in PBS, and then washed several times in PBS. Hearts were incubated in goat anti-mouse polyclonal secondary antibody (Abcam, # ab6669) at a 1:75 dilution for 1 h. The hearts were rinsed in several changes of PBS and mounted on slides in SlowFade® Gold antifade reagent (Invitrogen, #S36936). Three layers of fingernail polish were applied to the edges of glass coverslips to prevent damaging of the whole hearts. Antibodies conjugated with FITC were excited at 488 nm with an emission at 520 nm. The stained heart samples were scanned under a laser confocal microscope, Olympus Fluoview, equipped with a computer to record the images.

### qRT-PCR

Normal and mutant embryonic hearts at stage 36-37 were placed into 15 μl droplet cultures of Holtfreter’s solution containing antibiotics [1]. Mutant hearts were placed in droplets containing 7 ng/μl of RNA derived from clone #291 and incubated at 14°C for 72 hours. Each treatment group consisted of 10 hearts. RNA was extracted using a NucleoSpin RNAII Kit (Macherey-nagel) from ten mutant hearts treated with the active clone of human RNA (microRNA-499c), from 10 untreated (treated only with lipofectin) hearts as a control, and from ten normal hearts. qRT-PCR was performed with a Rotor-Gene machine using a Rotor-Gene SYBR PCR kit (Qiagen #204074) with primers as reported in Table 1[1]. Expression in normal heart was considered as 100%. Expression was normalized (divided) by β-actin expression.

**Table 1:** Primers used for genes in real time RT-PCR experiments

**Gene of interest**	**Forward/ Reverse**	**Primer**
Tropomyosin	Forward	5^′^-ggagcttgaccatgcgctgaa
Tropomyosin	Reverse	5^′^-tgagaaccgacacaaagcaagagg
troponin T	Forward	5^′^-ccaagggcttcaccgggctcaa
troponin T	Reverse	5^′^-tggcagaggtggaatggatcacagg
α-syntrophin	Forward	5^′^-ggactctccaccgcctccctctc
α-syntrophin	Reverse	5^′^-ccccgcttcatccttcgctctga
β-actin	Forwar	5^′^-tccatgaaggctgcccaact
β-actin	Reverse	5′-tggcgccacatctgattgat

## Results and discussion

On the basis of our results we hypothesize that normal human fetal heart expresses an RNA, which is a functional homologue to the axolotl MIR, and which probably is required for human heart development and function. Our results have clearly shown that if we clone this RNA from human fetal heart and transfect it into mutant axolotl hearts, normal heart development is restored. In an earlier publication, our laboratory showed that RNA extracted from human fetal and adult hearts, but not from skeletal muscle, rescued the development of mutant axolotl hearts in organ culture [3]. These earlier experiments suggest that total human heart RNAs, but not skeletal muscle, contain functional homologues of the MIR (myofibril-inducing RNA) derived from normal embryonic axolotl anterior endoderm [1].

**Figure 1:**
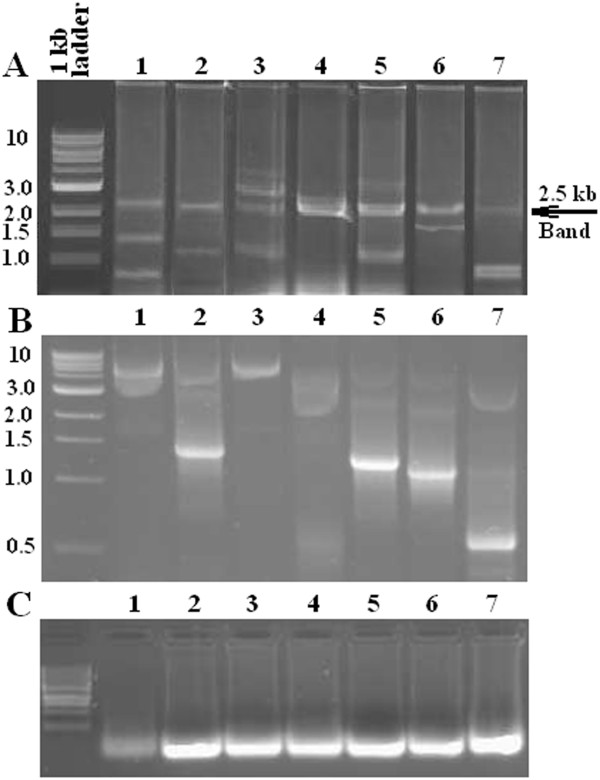
**Successful cloning of human RNAs.** Gel electrophoresis of (**A**) individual plasmid digestion fragments with BsrGI. A uniform 2.5 kb band represents the unchangeable vector portion, and variable bands are different size cloned RNAs. (**B**) Gel electrophoresis of cloned DNAs from PCR. (**C**) Gel electrophoresis of cloned RNAs from reverse transcription reaction.

A cDNA library was generated from total RNA extracted from human fetal heart that was purchased from Agilent Technologies Inc (Santa Clara, CA). We randomly cloned 400 individual RNAs from human fetal heart using the pDONR222 plasmid as a vector. DNA clones were synthesized by PCR using the vectors as templates and M13 primers. The PCR products were visualized through agarose gel electrophoresis and ethidium bromide staining. A vast majority of clones showed unique DNA bands (Figure 1B) indicating the presence of specific DNA inserts in the plasmids (Figure 1B). The RNAs were synthesized by using an *in vitro* transcription reaction and run on an agarose gel to test the result of the reaction (Figure 1C). RNA clones along with 0.1 mg/ml of lipofectamine transfection reagent were diluted to a concentration of 7 ng/μl in Holtfreter’s solution containing a physiological mixture of salts required for cardiomyocyte contraction. The hearts were incubated in a plastic container at saturated humidity and room temperature. Each individual clone was tested on three hearts.

**Figure 2:**
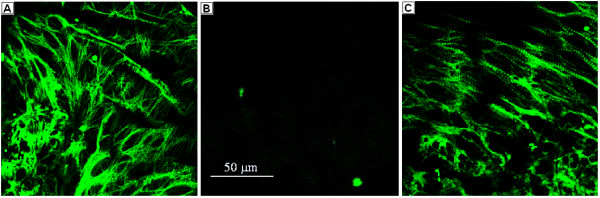
**Tropomysin expression in RNA-treated mutant axolotl hearts revealed by immunofluorescent staining with confocal microscopy.** (**A**) Normal heart. (**B**) Mutant untreated heart. (**C**) Mutant heart treated with cloned RNA (clone #291) after 5 days of incubation in organ culture. Magnification is the same in all images.

Transfection with RNA derived from clone #291 was found to induce mutant hearts to beat. Initially, the treated hearts beat sporadically. However, with additional time in organ culture, the beating became more vigorous and regular. Hearts were fixed in paraformaldehyde and stained with monoclonal anti-tropomyosin antibodies. Confocal microscopy revealed significant expression of tropomyosin in hearts treated with RNA derived from clone #291 (Figure 2C). Mutant axolotl hearts, which were not treated with any RNA or with RNA derived from non-active clones, do not show tropomyosin expression and beating (Figure 2B).

**Figure 3:**
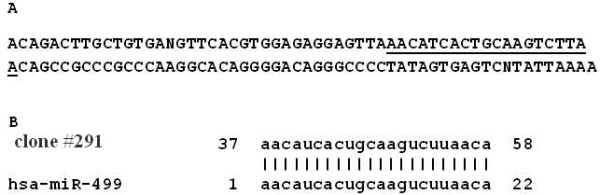
**(A) DNA sequence of cloned RNA from the clone #291 (microRNA-499c).** Underlined area is 22 bp microRNA-499. (**B**) Alignment of cloned RNA with microRNA-499 generated in Mirbase [8].

**Figure 4:**
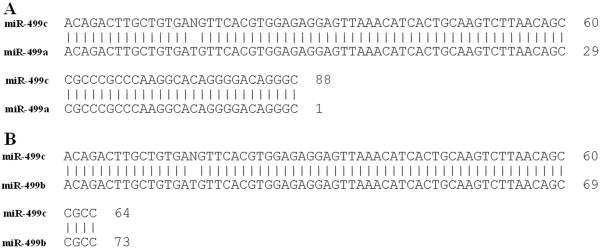
Alignment of cloned RNA (microRNA-499c) with (A) microRNA-499a and (B) microRNA-499b.

The sequence analysis revealed a 111 bp long sequence of the cloned RNA (Figure 3A). We screened the sequence in Mirbase [8] and found that 22 bp microRNA-499 fragment is included in the cloned sequence (Figure 3B). Screening of the human genome database with the Basic Local Alignment Search Tool software (BLAST), on the NCBI website showed that the cloned sequence from clone #291 is identical to an intron of one of the myosin heavy chain (MHC) genes. Also we screened the online microRNA database, Mirbase [8], and we found homology with a 22 bp miR-499 fragment included in the cloned sequence (Figure 3B). There are two known forms of the microRNA-499 precursor: microRNA-499a and microRNA-499b. Alignment of the cloned RNA, microRNA-499c, with precursors microRNA-499a and microRNA-499b showed that although they differ in size, they have common and overlapping sequences (Figure 4B). Precursors of the microRNA, microRNA-499a has 122 bp, while microRNA-499b has only 73 bp and the newly discovered form, microRNA-499c has 111 bp. Alignment showed that a new form of microRNA-499c has 88 common bp with microRNA-499a and 66 common bp with microRNA-499b. Thus, we conclude that we have discovered a new form of microRNA-499 precursor, which we term microRNA-499c.

### Time-course experiment

**Figure 5:**
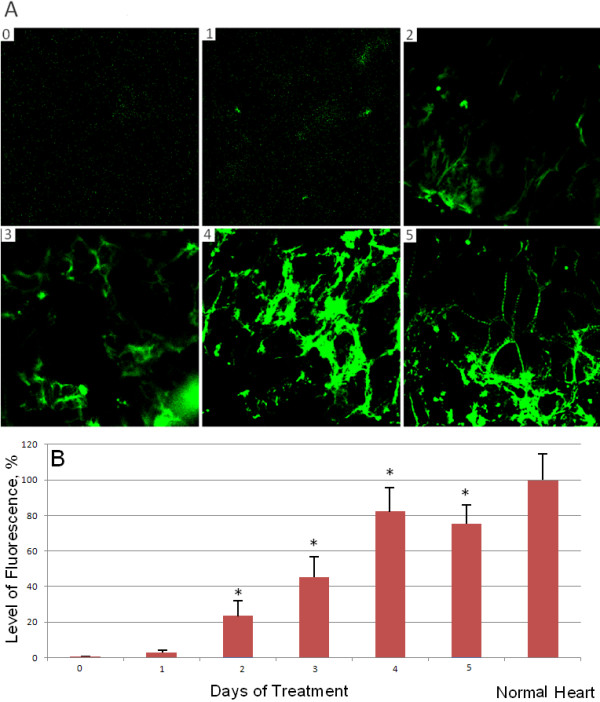
**Time-course study of the relative quantities of tropomyosin expression by immunofluorescence staining in RNA-treated (7 ng/μl of microRNA499c) axolotl hearts. A**. The immunofluorescent images show mutant hearts that were fixed and stained for tropomyosin after incubation with 7 ng/μl of microRNA499c for 0, 1, 2, 3, 4 and 5 days; the 0- control was without treatment. **B**. The average levels of fluorescence were quantified with ImageJ software as a percentage of that expressed in normal hearts, which was set to 100%. Significance (*p-value) between treated on the second day following days hearts and untreated hearts is *p* < 0.05, n=3.

The hearts from mutant axolotl embryos were incubated with the microRNA-499c during different periods of time: 1, 2, 3, 4 and 5 days. Embryos at the time of dissection were at post-heart-beat stage 36-37. After incubation with RNA, the hearts were fixed and stained with anti-tropomyosin antibodies (Figure 5A). Fluorecsence levels of heart images were quantified with ImageJ software (National Institutes of Health) (Figure 5B). At the initial point, (0 days) the heart does not show tropomyosin expression. During the 4 days of incubation, tropomyosin expression gradually increases. Starting with day 2 and following days, significance between treated hearts and untreated hearts is less than 0.05 (*p* < 0.05, n=3). Also, during the time increment of incubation, the numbers of striated myofibrils increases. Striated myofibrils are prominent by the 5^th^ day of RNA treatment of mutant hearts in organ culture, as well as in normal hearts (Figure 2A and C).

### qRT-PCR

**Figure 6:**
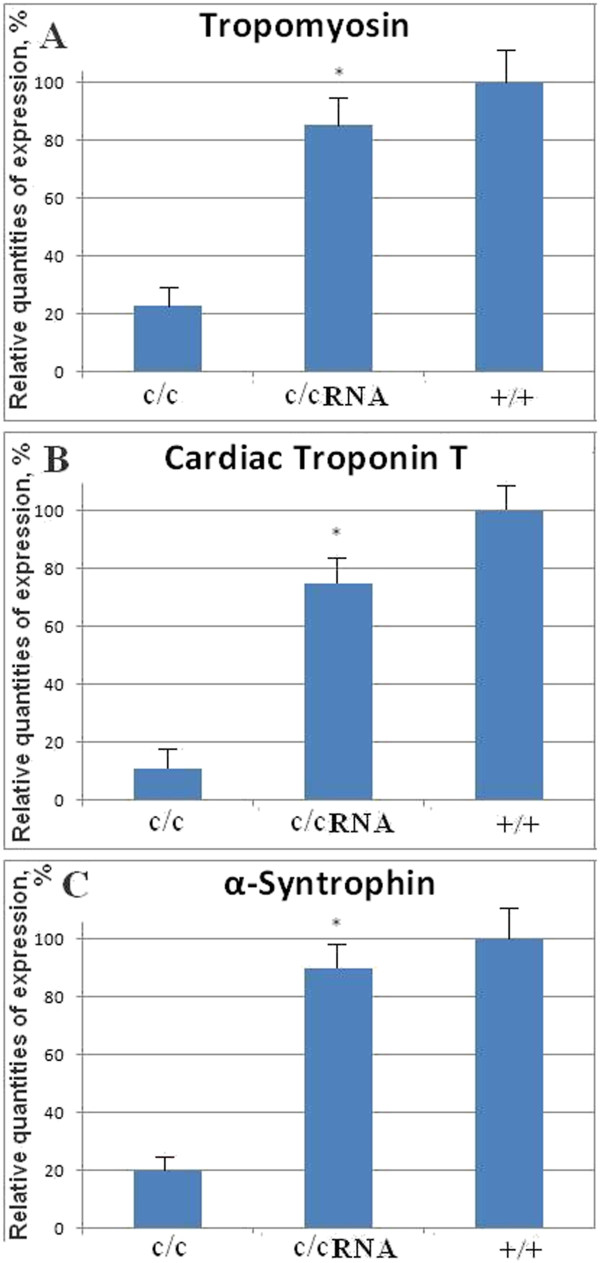
**Relative expression of cardiac markers by RT-PCR in mutant hearts (c/c), mutant hearts transfected with the microRNA-499c (c/c RNA) and normal hearts (+/+): (A) tropomyosin, (B) cardiac Troponin T and (C) α-syntrophin.** Expression was calculated as percentage of that expressed in normal hearts (expression in normal hearts was assumed as 100%). Significance between treated and untreated mutant hearts is for tropomyosin, *p* < 0.05, cardiac Troponin T *p* < 0.02 and α-syntrophin *p* < 0.03, n=10.

RNA was extracted from ten mutant hearts treated with the active clone of RNA (7 ng/μl of microRNA-499c), from 10 untreated (treated only with lipofectin) mutant hearts as a control, and from ten normal hearts. Expression of genes considered as cardiac markers included: tropomyosin, cardiac troponin T and α-syntrophin all of which increased significantly in comparison to β-actin in the RNA-treated hearts (Figure 6). Expression was calculated as % of expression relative to normal hearts, which was assumed to be 100%. In mutant hearts, expression of cardiac markers was much lower than in normal heart, as low as 10-20%. After treatment with the active clone of RNA (7 ng/μl of microRNA499c), expression in mutant hearts increased significantly up to 70-90%: tropomyosin – 75%, cardiac Troponin T – 70% and α-syntrophin – 90%. Tropomyosin is an important protein in sarcomere formation and in muscle contraction [9]. Troponin is required to regulate Ca(2+)-dependent contractions, and it was shown to be essential for sarcomere assembly in cardiac and skeletal muscles. The increased expression of these mRNAs suggests that the rescue of mutant hearts has taken place, when the muscle myofilaments start to be expressed and assemble into functional sarcomeric myofibrils. The increased expression of cardiac markers is consistent with data from our previous publication [1]. In that publication, expression of multiple cardiac makers: cardiac Troponin T, α-syntropin and tropomyosin increased after 36 and 72 hours incubation with Myofibril-Inducing RNA (MIR) from axolotl. In the current experiment, this active clone of RNA from human heart (microRNA-499c) also increased expression of cardiac troponin T, α-syntropin and tropomyosin.

Our results show that RNA cloned from human fetal heart has the capability of rescuing mutant axolotl hearts in organ culture bioassays. The rescue of mutant hearts was demonstrated by the development of beating in the hearts and expression of tropomyosin in organized myofibrils after incubation with the microRNA-499c. All three microRNA-499 precursors apparently originate from the same intron, which is initially the intron of the myosin heavy chain (MHC) genes.

The human microRNA-499 belongs to a family of micro RNAs encoded by the intron of the myosin heavy chain (MHC) genes, referred to as MyomiRs which also include microRNA-208a and microRNA-208b. MicroRNA-499 is highly conserved, being present in the genome of many vertebrate species (human, mouse, rat, bovine, Xenopus and zebrafish) [10]. Micro-RNAs are non-coding RNAs which regulate the translation of genes by binding to untranslated sites (UTRs) in their targets. Nascent precursor microRNAs are usually about one hundred or more nucleotides long, and later, microRNAs are split by enzymes into a short active 22 bp fragment.

In animals, expression of microRNAs occurs in two stages. First, in the nucleus, pre-microRNAs are cleaved from an extended primary transcript of the gene which then is exported to the cytoplasm of the cell. In the cell cytoplasm, endoribonuclease, called Dicer, cleaves the double-stranded RNA (dsRNA) into a short 22 nucleotide micro-RNA. The mature microRNA is incorporated into the RNA-induced silencing complex (RISC) which includes Dicer and other proteins. The miRNA binds complementary mRNAs and directs them to degradation by endonucleases or prevent their translation by holding them in the RISC. *In situ* hybridization analysis of mouse heart, brain, spleen, liver, lung, quadriceps muscle, kidney, and gut tissues shows that the mature microRNA-499 is abundantly expressed in cardiac tissue and almost absent in other tissues, including skeletal muscle [10]. Some of those predicted targets of microRNA-499 and microRNA-1 were shown to regulate cardiomyocyte differentiation. Transient transfection of microRNA-1 and -499 in human cardiomyocyte progenitor cells (CMPCs) reduced the proliferation rate and increased differentiation into cardiomyocytes. This effect most likely occurs by repression of histone deacetylase 4 or Sox6 because levels of these proteins are reduced [10]. It has been found that microRNA-499 promotes ventricular specification in differentiating human embryonic stem cells [11]. It was shown further that microRNA-499 expression increased in human stem cells differentiating into cardiomyocytes. Also, microRNA-499 transduction by the lentivirus of hESC-derived cardiovascular progenitors significantly increased the yield of stem cell-derived ventricular specified cardiomyocytes [11]. Other results suggest that expression of microRNA-499 in human cardiac stem cells (hCSCs) represses the microRNA-499 target genes Sox6 and Rod1, enhancing cardiomyogenesis *in vitro* and after infarction *in vivo*[5]. The level of microRNA-499 was 400 times higher in cardiomyocytes than in rat cortical stem cells. Cardiomyocytes derived from differentiation of hCSCs treated with microRNA-499 appeared to be larger, and their sarcomere striations were more evident [5]. Expression of microRNA-499 was observed to be increased a few hundred times during differentiation of human embryonic stem cells into beating clusters [4]. Also, overexpression of microRNA-499 enhanced expression of myocyte-specific enhancer factor 2C, the transcription factor which is involved in cardiac morphogenesis and myogenesis and vascular development [4]. Thus, microRNA-499 appears to play an important role during heart development, although the molecular mechanisms and the microRNA-499 pathways are not well understood at this time.

## Conclusion

Our results demonstrate clearly and unequivocally that human-derived microRNA-499c promotes the formation of cardiac myofibrils in cells of cardiac mutant salamander hearts and thus restores normal embryonic heart development in these lower vertebrate species. This observation is consistent with earlier publications showing that microRNA-499 plays an important role in cardiac differentiation and cardiogenesis during embryonic development, and it strongly suggests a ubiquitous and conserved mechanism of classic embryonic heart induction [12,13] and myofibrillogenesis across the spectrum of vertebrate species.

## Competing interests

The authors declare no competing interests.

## Authors’ contribution

LFL served as the Principle Investigator on the study and on the American Heart Association and NIH grants that supported the study. LFL and AK planned and oversaw all of the experiments and coordinated the research activities of the study. AK wrote the final draft of the manuscript. LFL edited the entire manuscript. AM and AK performed the bulk of the experiments while WL and JM helped with many experiments. MCH consulted, advised in molecular biology techniques and helped to interpret many findings. All authors read and approved the final manuscript.
